# Political Influence, Facilitators and Barriers in the Decision-Making Processes of Executive Nurse Leaders During the COVID-19 Pandemic in Spain: An Ethnographic Study

**DOI:** 10.1155/jonm/4761787

**Published:** 2025-11-04

**Authors:** Paula Alite-Cerezuela, Adelaida Zabalegui, Jone Velasco-Leon, Flores Vizcaya-Moreno

**Affiliations:** ^1^Nursing Studies Doctorate Programme, University of Jaume I, Castellon de la Plana, Valencian Community, Spain; ^2^Health Emergency Service of the Valencian Community, Valencian Community, Spain; ^3^Hospital Clinic of Barcelona, Barcelona, Catalonia, Spain; ^4^Department of Nursing, University of Barcelona, Barcelona, Catalonia, Spain; ^5^Donostia University Hospital, Osakidetza, Donostia, Gipuzkoa, Basque Country, Spain; ^6^Department of Nursing, Faculty of Health Sciences, University of Alicante, Alicante, Valencian Community, Spain

## Abstract

**Aim:**

To describe the political influence Executive Nurse Leaders (ENLs) in Spain had on decision-making processes during the COVID-19 pandemic, identifying facilitators and barriers to executive and policy-related decision-making and implementation.

**Method:**

A focused ethnographic study, with a direct qualitative analysis of manifest content. Twenty-five semistructured interviews were conducted with ENLs who held senior management positions in one of the executive roles included. The study includes the following executive roles: Chief Nurse Executive in the autonomous government and Chief Executive Officer, Chief Nurse Officer, and Assistant Chief Nurse Officer in healthcare organisations. The study period was from February 2020 until December 2022.

**Results:**

Three categories were identified: Category 1: Political influence—a description of the political involvement of ENLs and their influence on political actions, as well as the influence of politics on the work of ENLs; Category 2: Facilitators in the decision-making process—situational, personal and operative characteristics that were mentioned by participants as facilitating decision-making and implementation; Category 3: Barriers in the decision-making process—obstacles that reduced ENL influence in decision-making processes, and the barriers that were found when implementing decisions that were made.

**Conclusions:**

The political influence on the work of ENLs is discussed, as well as the influence of ENLs on healthcare policy during the COVID-19 pandemic. Facilitators and barriers during the decision-making process are identified. The study provides new knowledge to help understand the contribution and challenges of ENLs during the context studied. We recommend the implementation of governmental strategies to promote nurse leadership, to eliminate the detected barriers and to combat prejudice and gender bias, recognising the strategic value of nurses during healthcare crises. The findings need to be aligned with policy changes in countries with a similar political context.

## 1. Introduction

Promoting nurse leadership and their involvement in healthcare policy is a priority for the World Health Organisation (WHO) [1]. On an international scale, there are nurses in governmental positions with varying responsibilities and degrees of political influence [2], even those without direct influence on healthcare policymaking [3]. In Europe, only 18% of Ministries of Health have a nursing division, which limits their influence on healthcare policymaking decisions [2]. Historically in Spain, nurses have been a key part of the Ministry of Health. According to the governmental transparency platform, there are currently no nurses holding a senior official position in the Ministry of Health [4], and there is also no nursing division in its organisational structure [5]. However, the incumbent government currently has a nurse in a consulting role within the State Secretary of Health's cabinet, which also handles national coordination of the Nursing Care Committee [6].

Recently, the coronavirus pandemic underscored the need to invest in the professionals involved in the preparation for and response to any future crisis [1]. Nurses play a key role in all phases of disaster management [7] and in epidemic situations [8], and the coronavirus pandemic in particular saw a significant contribution from nurses [2, 8]. Despite their crucial role during the pandemic, nurses across the world felt silenced, with their concerns and interests neither seen nor heard [9].

There is a lack of nurse involvement in decision-making processes concerning healthcare policy, not only during healthcare crises but also in all areas of healthcare around the world [2, 10–13], as well as in Spain [14]. Public policy that does not take nurse perspectives of social issues into account may not be able to resolve these issues adequately [15]. Their absence during decision-making processes also impedes compliance with Sustainable Development Goals (SDGs) and Universal Health Coverage (UHC) [8, 11, 16]. Nurse involvement in the development of global health policy is fundamental in ensuring the effective expansion of healthcare programmes [10]. This lack of involvement in healthcare policymaking can be attributed to various causes [13, 17].

As such, the research team considers it relevant to analyse the experience and involvement of Executive Nurse Leaders (ENLs) in executive and policy-related decision-making processes during the response to the COVID-19 pandemic in Spain. The aim of the investigation is to answer the three following research questions:

• How did ENLs influence healthcare policy in decision-making processes during the COVID-19 pandemic in Spain, and in turn, how did policy influence ENLs in these processes?
• What factors facilitated ENL decision-making processes and implementation during the healthcare crisis?
• What barriers did ENLs face when making and subsequently implementing decisions during the healthcare crisis?

The aim of this work is therefore twofold: To describe the political influence of ENLs on the healthcare decision-making processes, they were involved in during the COVID-19 pandemic, and to identify facilitators and barriers perceived by ENLs when making and implementing decisions during their response to the healthcare crisis.

## 2. Materials and Methods

### 2.1. Design

A focused ethnographic approach was taken [18, 19]. This design allows for the study of a specific issue and the shared experience of a group in particular contexts and for the identification of the characteristics, behaviours and common experiences found therein [18]. This method was chosen as the present study examines a specific topic that is limited to a particular time period and concerns a very targeted population [19]. As such, the method was deemed appropriate to identify the political influence of ENLs during the COVID-19 pandemic in Spain, as well as the barriers and facilitators they faced.

### 2.2. Recruitment of Participants

In order to have a sample that was representative of the entire country, a mixed sampling method was applied, combining the snowball, convenience, purposive and maximum variation sampling methods [20]. Study participants were nurses holding one of the senior management roles included in the study during the study period (February 2020 until December 2022). Participant inclusion and exclusion criteria are shown in Table 1.

In Spain, healthcare powers are devolved to the Autonomous Communities (ACs) [21]. This transfer of powers means there are organisational differences between autonomous governments. For example, there are differences in the organisational structure of senior governmental positions, which means there are some positions that exist in certain ACs but not in others. As such, to better compare results between ACs, the research team decided to sort participants into one of four executive positions, as recognised in international literature. These positions are as follows: Chief Nurse Executive (CNE) [22], Chief Executive Officer (CEO), Chief Nurse Officer (CNO) [22] and Assistant Chief Nurse Officer (ACNO). The term ENLs is used by the authors to refer to all study participants.

Participants were sorted into their respective executive category as follows: CNE encompasses ENLs that hold a senior administrative role in the Ministry of Health of the Autonomous Community (MoHAC) or in the organisational structure of the healthcare system of their AC. The CEO category is for individuals with the highest seniority overall within the healthcare organisation itself (e.g., the hospital), whereas the CNO category is for individuals with the highest seniority among nursing staff in the healthcare organisation. Finally, the ACNO category includes ENLs that hold a less senior role, but who are still part of the healthcare organisation's executive committee.

### 2.3. Data Collection

In order to gather more details on the experiences and perspectives of interviewees and establish the meaning they attribute to them, in-depth semistructured interviews were conducted [23]. Interviews were conducted online using the Microsoft Teams platform. The interview script is available as a Supporting file (available here).

The majority of interviews lasted less than 60 min, the shortest lasted 39 min and the longest lasted 118 min. Interviews were conducted by Flores Vizcaya-Moreno, Adelaida Zabalegui and Paula Alite-Cerezuela.

After the session, the interviewer created a literal transcription of the responses provided. To preserve participant confidentiality and anonymity, any references to cities or names of healthcare facilities were removed from the transcript. Participants were assigned a random code preceded by the letter M (male) or F (female). The transcription was reviewed by Jone Velasco-Leon. This collection period lasted from June 2022 until June 2023. The sampling and data collection processes were dynamic and concluded only when theoretical data saturation and redundancy of data were reached [24].

### 2.4. Data Analysis

In order to ensure a systematic data analysis process, qualitative content analysis was used [24]. The 16-step method proposed by Assarroudi et al. was used [24] to conduct a directed qualitative content analysis. Step 9 differed from the proposed method, with the categories being developed inductively instead of theoretically, as these arose from the data being analysed [25]. This was done in order to be able to answer our research questions. To avoid discrepancies in the analysis process, the categorisations were pretested, thereby increasing the reliability and credibility of the study [24]. Preliminary data coding was performed inductively, sorting responses into the established categories according to their meaning [24]. The units of analysis were transcribed interviews, as manifest content analysis was conducted [24].

### 2.5. Rigour

The criteria of confirmability, credibility, dependability and transferability were applied to guarantee quality of study [26]. To ensure confirmability of data, participants were permitted to review, modify or correct their transcriptions. Seven interviewees made minor changes in order to clarify their responses. After the data were analysed, participants were then provided with a summary of the results to review (member checking) [27], thereby ensuring that their perspectives were adequately represented [23]. No negative reviews were recorded, with one single observation detailed later in the results section.

Credibility of findings was reinforced through critical reflection of investigators on their potential bias, which allowed for continued objectivity throughout the research process [27]. Literal transcriptions of the interviews were created and subsequently reviewed independently by a second researcher, ensuring complete immersion in the data.

Dependability was achieved by providing an exhaustive description of the methods used [27]. Using a structured, 16-step method increased rigour during the data analysis process [24]. Furthermore, reliability was further supported by independent coding of data by two researchers [28].

For ensured transferability, the article details sampling strategies, the criteria for participant selection and the context under study [27].

A total of 54 literal quotations were used as a base for the main codes presented in the results. Another 49 additional quotations are provided. This strategy ensures inclusion of quotations from various participants, increasing transparency and trustworthiness [29].

Finally, the SRQR checklist was applied [30], which is internationally recognised for use in reporting qualitative research (Supporting file).

### 2.6. Ethical Considerations

Ethical approval for the study was given by the Human Research Ethics Committee (CEISH) at Universitat Jaume I in Castellón de la Plana, Spain. Reference CEISH/93/2023.

All interviewees gave their written consent to participate in the study. Recordings and transcriptions of all interviews will be kept on a virtual drive and are the property of Universitat Jaume I.

## 3. Results and Discussion

### 3.1. Participant Characteristics

Of the 59 participants invited to take part in the study, 25 participants accepted the invitation (a response rate of 42.37%). After analysing participant discourse, researchers excluded one interview after finding that the participant only referred to her work in a Ward Manager role, which was an exclusion criterion in this study. The final sample comprised 24 ENLs from 13 of the 17 AC in Spain.  Table 2 details the sociodemographic characteristics of the participants.

### 3.2. Results

**Figure 1:**
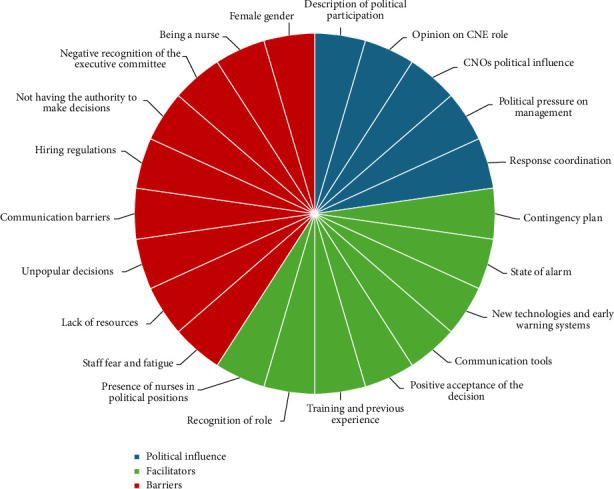
Code-category distribution.

The results are split into the three categories previously defined by the research team to ensure that the study aim is achieved.  Table 3 includes additional quotations from participants that support the presented findings.  Figure 1 shows a graphical representation of the distribution of codes per category.

#### 3.2.1. Category 1: Political Influence

This category includes codes describing the relationship between ENLs and the political sphere, as well as their involvement therein. It also covers the dynamics of influence, both that of ENLs on political decisions, as well as that of politics on the work of ENLs.

##### 3.2.1.1. Code 1: Description of Political Participation

This code describes ENL participation in decision-making within the autonomous health authority. All participants agreed that ENLs should hold senior executive positions in this area.

It must be highlighted that each AC has its own organisational structure with regard to its healthcare system, which creates a discrepancy in ENL competencies and functions across the various regions.

*I hold the most senior nursing role in my [autonomous] healthcare system […]. Comparing ACs is a little complicated, […] as their system isn't set up like ours is. F15*

In some ACs, there are no ENLs holding senior roles within the MoHAC. In these cases, ENLs in said regions express the need for nurses in positions of autonomous political leadership.

*We've definitely been missing a nurse's input [a CNE in the MoHAC] like other autonomous communities have […]. It could have helped us during the pandemic, having a nurse's perspective in that [decision-making] process, […] we still don't have that […] [decision making] in politics […] I think having that representation is really important […] nursing staff could bring a lot to the table. F24*

##### 3.2.1.2. Code 2: Opinion on CNE Role

There are various opinions on the need to establish a specific CNE role within the MoHAC that is dedicated to the management of nursing personnel. While some participants consider this role to be vital, others feel that ENLs should be more integrated into existing organisational structures within autonomous healthcare politics, with no need for a specific nursing-related executive role.

*If only there were no need for a subdivision of nurse managers [CNE role] and that nurses were equally valued for their management capabilities. There are some nurses who are suited to it and some who aren't, […] I also don't think that you should be able to move into any other role just by being a nurse in the first place; not a nurse, not a doctor, not a pharmacist […] Does there have to be another level of care management [a CNE role]? I'm not so sure. But do there have to be nurses? Yes. F15*

*I'll always think that another level of caregiving management [CNE role] would be a good decision because, […] we're moving towards an ageing population where patients need ongoing care, they already receive a diagnosis and treatment. So, for a healthier future, we need to focus on the promotion of health and on prevention. What is missing is nursing care. F17*

##### 3.2.1.3. Code 3: CNO's Political Influence

Some CNOs enjoy an increased level of independence and have access to direct communication channels with political representatives, which allows them a greater degree of influence on political decisions.

*I'm in touch with someone in our [autonomous government's] healthcare department and I said […] I offered our hospital's help with vaccinations. […] When I got to work, I said to my CEO, […] who has complete faith in us and our abilities, […] ‘Whatever you think,' [he said]. Not even two hours later, we were with the president of the autonomous community looking at sites where we could do the vaccinations. F5*

However, there are also responses from CNOs in the opposite situation, who have no political influence whatsoever.

*I had no weight, politically speaking. Maybe I should have listened a bit more or taken some things into consideration. But that's politics for you. F18*

##### 3.2.1.4. Code 4: Political Pressure on Management

ENLs described the political pressure they felt, explaining how they complied with decisions that they did not completely agree with, be that due to ethical concerns, perceived inefficiency or because they involved increased pressure on the healthcare system that led to increased-risk situations.

*Some decisions were imposed by the Ministry [MoHAC] or from the Government, […] if you are part of management, you also have to stick to the rules coming from above, even if you don't completely agree with them […]. Political decisions that were made without proper scientific evidence. Maybe the public health sector was saying one thing but then the political side carried more weight. F18*

*Public image and politics really clash with the hospital's interests. I found myself in several quite tense situations, because I saw that reducing staff so much was causing many more errors with medication than on previous days. F20*

However, to a lesser extent, some ENLs did not experience negative political pressure, and furthermore, they considered they were trusted when it came to their handling of the pandemic.

*During the pandemic, we always felt supported by the Ministry [MoHAC]. They always had total faith in us. We would say: ‘we need to take this approach' and they would take that approach, with no political involvement whatsoever. M3*

##### 3.2.1.5. Code 5: Response Coordination

To provide a solid, cohesive response to the pandemic, interdepartmental links were strengthened, often leading to new relationships between AC ministries.

*Working with the police to establish mandatory isolation periods, mandatory lockdowns […]. We had several meetings with the Ministry of Education [of AC] and oftentimes, when we had to close a classroom or even a full school, for example, […] we had to meet with the [AC] Vice Minister of Health and also with the [AC] Vice Minister of Education, […] the Head of Public Health. F16*

*Having [Nurse] Coordinators acting as the bridge between the Health and Social sectors, or between Health and Education […]. We had to create these links, we couldn't keep working on our own […], there have to be communication lines and there has to be infrastructure in place for this coordination. F2*

#### 3.2.2. Category 2: Decision-Making Facilitators

This category includes situational, personal and operative characteristics identified by interviewees as facilitating factors in decision-making processes during the COVID-19 pandemic.

##### 3.2.2.1. Code 6: Contingency Plan

Having a contingency plan was highlighted as a facilitator for decision-making processes, as it lays out predetermined actions to be taken in line with the epidemiological situation in question. Plans such as these also took potential critical situations that could arise into account. In the interview responses gathered, it is unclear whether a specific plan for potential epidemiological emergencies existed before the start of the pandemic or not.

*Having a contingency plan helped us to plan our next step and predict the needs that would arise in each situation we faced. F14*

*Designing the […] contingency plans […] helped us know how to react to a rise in cases during the pandemic, how to anticipate our next steps and reorganise our infrastructure more efficiently for subsequent waves. F10*

##### 3.2.2.2. Code 7: State of Alarm

The Spanish government's declaration of a state of alarm allowed for more flexibility in bureaucratic and administrative processes. This increased flexibility was a key facilitator in decision-making processes during the pandemic.

*The state of alarm gave us a huge amount of leeway. You'd have to write up a justification report and almost everything was valid. M3*

However, interviewees noted that this situation did not continue indefinitely.

*CEOs were given an unprecedented level of autonomy […] that we'd never had before, and now we've gone back to not having it […]. Making legislation more flexible […] let us make decisions that we'd never been able to make before, for example, scheduling staff who were on unpaid leave […], postponing holidays, allocating highly unusual shift patterns […] that flexibility is also what helped us survive. F4*

##### 3.2.2.3. Code 8: New Technologies and Early Warning Systems

This is one of the most frequently mentioned facilitators by interview participants. New technologies played a fundamental role in healthcare strategy development during the pandemic.

*Everything with remote healthcare, setting up remote appointments, video calls. All that was very strategic because those systems remained in place afterwards. […] we started right in the middle of the pandemic, and I think it was really important to be able to continue providing care. F24*

*[At the COVID Call Centre,] we involved artificial intelligence at vaccination times, […] for appointment-related things […]. It was a robotic call handler that could take 4000 to 5000 phone calls at once, answering different people, and we were training it as we went along. M6*

These technologies were also used to provide up-to-date information to workers, whether in the form of online training, documents shared on official websites or online conference calls.

*We didn't use ZOOM [videocall software] before. We did have some kind of platform, but everything to do with online communication and training got so much better. F15*

The various early alert systems provided real-time epidemiological information, allowing for predictions of how the pandemic would progress, and as such, more informed planning of healthcare services to cover future healthcare needs. Many of these alert systems were designed on an ad hoc basis to analyse the behaviour of the coronavirus.

*We were working with mathematicians from the university to make predictions. […] it gave us an idea of where the pandemic was headed. Well, at first it was very far off, but it became really refined later on. So, we knew that cases were rising and that we were going to need 150 beds. F23*

##### 3.2.2.4. Code 9: Communication Tools

WhatsApp and personal interactions between ENLs helped to gather information about how other organisations were handling the healthcare crisis.

*Using WhatsApp […] we would ask each other: […] ‘How do you do appointments? […] How are you running your ICUs? Do you have resources?'. When you had a question, you always went to see how other people were doing it. F23*

There were also various measures implemented to improve staff communication, whether that was communication with superiors or to update staff on the organisation's current situation.

*Setting up […] meeting points […] for supervisors with their staff to discuss matters, give encouragement, etc. […] [With] the supervisors […] we'd talk about the current situation, […] we'd try to encourage them to talk as well, and I think that helped […] we still do it […] it was well received and is still thought of positively. F14*

*I was concerned about being present and giving explanations. Because sometimes the staff wouldn't understand the steps being taken and they'd say, ‘So now we need to set up another unit?'. Yes, we do, and we need to do it in 6 h. People really struggled to understand the reasoning because their experience was different, so that's why getting that information was so important. F8*

##### 3.2.2.5. Code 10: Positive Acceptance of the Decision

The opinion and acceptance of those putting the decisions into effect facilitated their implementation.

*People saw it almost like humanitarian aid, where you had to help out however you could […]. At the beginning, everyone wanted to help, and they saw it as something they just had to do, and that was a good thing, it really made things easier for me. F20*

*When […] there was no-one that I could swap in, I'd send an email to the nurses. I'd tell them about the situation, thanking them for their work and asking if anyone would volunteer for more shifts because we were short-staffed. The next day […] management called me saying, ‘What happened? All of the nurses are phoning in saying, ‘Count me in.' F12*

##### 3.2.2.6. Code 11: Training and Previous Experience

Previous training in management is considered a facilitator for increasing ENL participation in executive senior official roles.

*I think training in healthcare management should be better regulated, as we can only bridge that gap holding us back from positions of decision-making [authority] by having a solid education behind us. F10*

Hospital organisations with previous experience in handling severe infectious diseases and/or in catastrophes resulted in much better prepared teams who were able to provide the appropriate response to the pandemic.

*This hospital is one of seven reference centres for Ebola, […] we were well versed and well informed to take on a healthcare crisis. F5*

ENLs with years of experience in the role are familiar with how the system functions and may have also faced other critical situations before, so decision-making in crisis situations is easier for them.

*My professional life has always involved tough decisions. […] I had to make very important decisions during the Ebola [outbreak]. […] in a terrorist attack […]. I've had the chance to make, shall we say, ‘weighty' decisions […]. Because of my experience, thankfully, it prepares you to face the situation and not crumble, at least. I mean, you have to be there to lead your amazing team. F21*

##### 3.2.2.7. Code 12: Recognition of Role

Feeling recognised both by superiors and by colleagues is considered a facilitator by ENLs. This recognition gives their involvement the same weight, or value, as chief officers in other professional fields, meaning their suggestions are listened to and they have a stronger influence on decision-making processes.

*The role of Head of Nursing [CNO, my role] is on the same level as any other chief medical officer or on the organisation's executive committee. M22*

*I have a place, and I think I have influence, and my boss respects me. F19*

ENLs are aware that this recognition is not always guaranteed. One participant only accepted the role under the condition that their role would be recognised as being on the same level as other chief executives.

*One of my conditions for accepting this position was that I had to be on the Executive Committee, on the same level as the rest of the Directors [chief executives]. The then-CEO, […] said yes, that she thought that was appropriate. F9*

##### 3.2.2.8. Code 13: Presence of Nurses in Political Positions

During the pandemic, there were nurses holding the positions of Minister of Health and Vice Minister of Health in two ACs. All ENLs from these ACs who were interviewed noted that a nurse holding this most senior role was a facilitator for ENLs to also hold senior official roles, as well as for making their suggestions be heard and valued.

*[If the person in the most senior political position] hadn't been a nurse, I think it would have been much harder for me to have gotten this role, a CEO position […] It just wouldn't have happened otherwise. F4*

*We were lucky, […] and it's because [the person in the most senior political position] was a nurse. She understood us. F1*

#### 3.2.3. Category 3: Decision-Making Barriers

The majority of the decisions mentioned by ENLs were unanimous decisions, shared with their organisation's respective executive committee. In the following section, we detail the barriers identified by ENLs that reduced their influence in decision-making processes, as well as the barriers to implementing the decisions that had been made.

##### 3.2.3.1. Code 14: Staff Fear and Fatigue

One of the problems faced when implementing decisions made by ENLs concerned the physical and psychological wellbeing of staff carrying out the actions, with ENLs often having to put supportive measures in place for staff and having to help manage fear.

*I had to handle lots of staff anxiety attacks, […] because of how anxious they felt going into a room with a COVID patient, their fear of becoming infected, of taking the disease back home with them. F18*

Fear of infection also impacted the management of personal protective equipment (PPE) resources, be that either because a lack of resources heightened staff fear or because staff fear led to improper and/or exaggerated use of PPE.

*There was a lot of alarmism at that time […] you'd find places where they'd use, surgical mask, FFP2 masks, the gown, apron, cap. I think there was a lot of fear and that made you want to protect yourself […]. And back then, that fear made us use more resources. Understandable, but obviously more challenging from a [resource] management perspective. F17*

As the response to the pandemic progressed, staff began to experience burnout, with interviewees reporting high levels of fatigue. Some ENLs feel that the pandemic left a lasting mark on healthcare staff's disposition, which has persisted over time.

*Things need to be done, people are tired […] it would have been so easy to just give people holidays, three days off. […] a way to look after the ones looking after others. There was no strategy in place to look after healthcare staff. […] Not feeling cared for is demotivating, and that still has lasting effects today. F19*

##### 3.2.3.2. Code 15: Lack of Resources

The lack of resources across the board also impacted the implementation of decisions that had been made due to not having the means to do so.

*Yes, there are some decisions that couldn't be made for logistic reasons, care-related reasons, and a lack of human resources and materials. M7*

*To properly care for patients, we needed more staff, we needed more materials, and we needed to make quick decisions faster than COVID could take root and spread. F16*

##### 3.2.3.3. Code 16: Unpopular Decisions

The possibility of decisions being received poorly by the public made it difficult for ENLs to implement them.

*We were under a lot of pressure […] Difficult decisions, unpopular decisions, […] you didn't want to irritate people, it felt terrible having to put a stop to certain activities where nothing could have even gone wrong, because we just didn't have data […] that went beyond theoretical situations we could come up with. M13*

Negative perceptions of other departments also played a role in ENLs implementing decisions that were deemed necessary. Having the opposite opinion to the preventive medicine department was mentioned three times.

*We started testing in patient units. Because, at first, we didn't do PCR tests on patients at admission […]. We started doing that with input from the Occupational Health and Safety Department and with Preventive Medicine, but sometimes we disagreed. And obviously the Preventive Medicine Department has to [assess the situation] […] but in the end you disagree, and you do what you think makes more sense strategically. It definitely creates uncertainty. F24*

Mirroring the facilitator of positive acceptance of decisions, a barrier for management was negative criticism from staff about the decisions that had been made.

*The decisions that were made here were always under scrutiny. No matter what decision you made, there would always be someone unhappy. So, you had to be extremely prepared because your intentions were always good and were based on what you had in front of you. […] There was an emotional burden, even a feeling of guilt, at times. F17*

##### 3.2.3.4. Code 17: Communication Barriers

The lack of information about the new disease, as well as the ever-changing protocols and strategies of healthcare authorities, was a barrier in decision-making processes. This situation forced ENLs to make decisions despite extreme uncertainty. At times, there were conflicting or contradictory answers given during the pandemic response.

*Sometimes I think [we were] a little uncoordinated. At that moment in time, I think society expected coordination from us […]. I think that even the protocols themselves were a little uncoordinated at times. ‘How come yesterday [self-isolation] had to be 14 days, today it's 10 days and tomorrow it'll be seven? What's going on? I'm not buying it.' F17*

There were several other barriers to effective communication. It is considered that the communication channels used in the healthcare system are not efficient enough. Furthermore, it was mentioned that there were some CNOs with no direct lines of communication with the MoHAC.

*The communication channels aren't as fast or effective as they should be. M11*

*A field hospital was set up and I think we only found out after seeing it on TV. We were never able to make use of it, and we didn't even know we could send patients to it. F12*

Some decisions were very exposed to political criticism and pressure from the media. This exposure had to be kept in mind during decision-making processes, often acting as a barrier thereto.

*On a wider scale, decisions like these are very politically charged and [draw] a lot of media attention, so you were scared of making mistakes. F16*

*There are times where the consequences [of our decisions] clash with political issues or where stories in the press clash with the reality of the situation. If you want to look good to the press saying I'm the one administering the most vaccines, good for you. But later, [when] your staff is reduced because we needed more people in the inpatient department after another wave, it's fine for you to say […] this week I'm going to administer […] [fewer vaccines] and there were some really quite heated moments. F20*

##### 3.2.3.5. Code 18: Hiring Regulations

Hiring regulations differ between ACs, with some ENLs believing the hiring system is obsolete. They also consider it a barrier to the recruitment and retention of trained, experienced staff.

*One downside of organisations, […] is the hiring system, the different [hospital] management teams can't be competing with one another, we can't be poaching staff from one another. F5*

*Hiring regulations, which are very strict, and you can't base it on your experience or knowledge, but rather on the number you are on the list. F23*

##### 3.2.3.6. Code 19: Not Having the Authority to Make Decisions

The ENLs who were interviewed held senior official roles relating to management, but certain decisions, which they felt were the right ones to make, could not be made because they were outside the ENLs realm of competence.

*When they were going to open the [field hospital]. To me it was like robbing Peter to pay Paul [solving one problem to have the same issue at another site]. But anyway, I had no say or vote in that regard. F21*

*We usually worked with single isolated patients, which is standard procedure […]. Then, when such a huge influx [of infected patients] arrives, having people donning and doffing [PPE] constantly is terrible […]. We had never dealt with isolation wards involving [isolating] an entire unit, where once you enter the unit, you can move around each different [area] […]. Changing [isolation protocols] […] Who oversaw that? Someone somewhere thought that it wasn't my place to make those suggestions. F15*

##### 3.2.3.7. Code 20: Negative Recognition of the Executive Committee

While this code is related to Code 19, the difference here is that ENLs consider that the obstacle to them making decisions they deemed to be correct was not that the decision lay outside of their area of expertise but rather because of a negative response from management: be that not being deemed capable enough by the rest of the executive committee or perhaps not being listened to by superiors.

*Complete involvement. Taking our opinion into account is another story. We've not always been listened to. Your opinion was considered or not depending on the topic […]. I felt very alone, they only cared about opening more units, they didn't care whether there were staff available or absent, that was my problem. They paid us little attention. F8*

*There was also some tension within the management team […] other managers [chief executives] felt like maybe they were the ones who had to be [in charge of that area] […] it was decided by the CEO, I think because he thought I was the right manager to be in charge, because everything fell back onto nursing staff: if there was a PCR to do, it was Nursing's job; if a vaccination plan had to be made, it was Nursing's job. So, he thought that I was the person who could take on all those responsibilities. F2*

##### 3.2.3.8. Code 21: Being a Nurse

Another barrier to holding a position on the senior management board is the concept of nursing being inferior to other disciplines. Within the executive board, being a nurse is considered a barrier, as ENLs feel that their suggestions or opinions are not always taken seriously due to their background as a nurse.

*I'm convinced that many of these decisions, or at least some of them, weren't followed through because the one who came up with it was a nurse. I'm positive that if I were, let's say, a doctor, my ideas would have been taken more seriously, I'm sure of it. M13*

*The old clichés come back into play. So, when you're a nurse or when you manage the Nursing Department, it seems like your area of expertise or what you can handle is set in stone […]. There need to be nurses in more areas. Especially in management […]. Being a nurse shouldn't hold you back. And I think it still does. Because that person's job title is looked at rather than the skills they have. F15*

##### 3.2.3.9. Code 22: Female Gender

Two female informants highlighted that, in addition to being a nurse, being female is also a barrier, intensifying discrimination in decision-making processes at the executive levels under study.

*I've really had to argue my case, not just because I'm an executive and a nurse, but also because I'm a woman. F2*

#### 3.2.4. Participant Feedback on Results

The summary of results that was sent to participants received zero objections. Only one female informant wished to stress that, as an ENL, she felt that being female was not an obstacle to her involvement in decision-making processes. In this regard, as the code is backed by textual quotations from other interviewed women, the authors do not consider it to need modification. However, to maintain rigour in the study, the participant's observation is included here in this section.

## 4. Discussion

This qualitative study analyses the political influence of ENLs in decision-making processes during the COVID-19 pandemic in Spain. It also identifies the facilitators and barriers highlighted by ENLs when making and subsequently implementing decisions during the healthcare crisis.

The national context in question, with 17 different Autonomous Health Systems, shows significant differences in terms of position, responsibilities, competencies and influence of ENL roles. This situation, also documented on an international level [2, 3], is particularly notable when it occurs in a single country. In Spain, assigning the role of CNE, CEO, CNO and ACNO is done by unrestricted designation by the autonomous government [31, 32], a senior appointed position filled at the discretion of the administration.

The findings indicated that the ability of ENLs to influence decision-making processes varies depending on several factors, such as respect from superiors or having a previous personal relationship. As such, establishing professional networks and strategic relationships with other influential figures is fundamental to leadership [33–35]. Nurses should be involved in scientific consultations during the design of public policies [14], thereby promoting evidence-based decision-making [1, 12, 36, 37]. Furthermore, their affiliation and involvement with political parties, unions and collegiate organisations is a key aspect of integrating a caregiving perspective into healthcare policy [14].

During the study period, there were two nurses in two autonomous governments holding the positions of Minister of Health and Vice Minister of Health [38, 39]. ENLs from these ACs feel that these circumstances facilitated their access to leadership roles. They also led to increased consideration and acceptance of their suggestions and ideas. Santillán-García highlights that, although their involvement may be mediated by their political affiliation, nurses holding political positions offer a caregiver perspective in the legislative framework, benefitting both the wider population and the profession itself [14].

Regarding the role of CNE in the autonomous government, there are some discrepancies between interviewees on the need for ENLs to lead a specific nursing department or whether, conversely, ENLs should lead other more general departments. The WHO recommends consolidate the role of Government Chief Nursing Officer (GCNO), granting them the power to define political priorities and introduce reforms in the nursing workforce, reduce inequality, and improve patient safety and service provision [2]. During the pandemic, some GCNOs played a vital role in national legislation and strategic planning [2]. For example, in the United Kingdom and Ireland, where GCNOs have direct access to policymakers, GCNOs were able to shape health policy by providing expert advice [2]. However, not all GCNOs were involved in national health policy decisions. According to the International Council of Nurses, only 41.5% of its 130 member countries had involved their GCNOs in national health decision-making. [40]. The lack of this role in Spain during the pandemic limited ENLs' influence and weakened coordinated responses.

In Spain, there were coordination issues across several areas: between the healthcare system and social services; in the mechanisms to share medical resources between regions; and ICU coordination on a national scale [41]. In this study, ENLs were found to have participated in coordination activities between healthcare organisations and also with other governmental departments, a function observed among some European GCNOs [2]. Coordination between healthcare organisations on a local and regional scale helped to share information and workers, allowing for role distribution across organisations and a common patient transfer system [42]. On an interdepartmental level, this coordination is paramount in providing complex healthcare responses involving several departments.

With regard to facilitators, having a contingency plan in place facilitated the systematisation of responses using pre-established decisions. The reviewed literature also mentions disaster plans as a part of organisational preparations for future crises [7, 43, 44]. In the studied context, the lack of pre-established protocols in care homes worsened the impact of the pandemic [41]. There is an urgent need to establish a national preparation and response plan for healthcare emergencies [41].

The ENLs that were interviewed stressed the importance of IT and early warning systems in predicting how the pandemic would progress in order to plan strategies accordingly. When the pattern of COVID-19 spread became known, this helped healthcare organisations to predict the number of potential new cases and future bed requirements—especially ICU beds. Although healthcare organisations already had early warning systems in place before the pandemic, these systems were modified to include COVID-19 case characteristics [45]. However, the assessment of Spanish systems was that they were weak, underscoring the need to improve them [41]. After the pandemic had begun, the influenza surveillance system was replaced by an integrated surveillance system for monitoring acute respiratory infections—including COVID-19—arising from primary care and severe acute respiratory infections arising from hospitals [46]. Nevertheless, there is still room for improvement, such as ensuring sustainable availability of mathematical modelling and establishing a systematic process for sharing data on testing and hospital capacity at a national level [46]. Furthermore, there is a need for the creation of a State Public Health Agency [41, 46], as these systems are currently managed by each respective AC. These improvements will be crucial to any response to future public health crises.

During a healthcare crisis, effective communication management is paramount [47]. Staff need clear instructions [48] and all necessary information must be readily available to them [47]. Our study has identified various channels used to provide information to staff, which aligns with results from Leppäkoski et al., which, among others, documented the use of the mobile application WhatsApp [48]. There are, however, questions about this application that remain unanswered, such as those concerning data security or staff being able to disconnect during nonworking hours.

There was a lack of information during the COVID-19 pandemic [49, 50], and decisions had to be made without proper evidence backing them [48]. This lack of information and the emerging evidence resulted in constant changes to guidance and protocols [50–52]. As such, ENLs had to make decisions despite a great deal of uncertainty, resulting in inconsistent responses. A lack of clear discourse can lead to tension [50]. Therefore, it is crucial that detailed communication plans be put in place for healthcare crises, such as providing specific channels for healthcare staff, setting up information hotlines for the public and even implementing artificial intelligence in call centres [41].

Positive acceptance of decisions by staff is a facilitator for implementing ENL decisions. Our study does not analyse the reasons behind staff acceptance of these decisions, but other studies have identified that education and previous experience in disaster management, as well as having a response plan already in place, increase the likelihood that workers can effectively manage a crisis [53]. Having faith in the organisation and their communicative actions also influence positive acceptance by staff members [54].

One particularly notable facilitator is having previous experience in disaster management, both for ENLs and also other healthcare staff, as well as having previous leadership experience. The literature supports encouraging ongoing education and training in biological risks and the use of PPE [41] and also in disaster management, both for staff [7, 55] and for nurses in leadership roles [56]. To increase involvement of ENLs in decision-making processes on both an executive and policymaking level, there must also be some degree of training in leadership [1, 2, 33, 35] and healthcare policy [2, 13, 17].

ENLs with more experience in management find decision-making during crisis situations easier, as they already know how the system works and have faced other critical situations before. As such, it would be beneficial to include these ENLs in executive and managerial positions [41] and for their inclusion not to be driven by political interests.

One of the main barriers was staff fear and fatigue. High levels of fear of COVID-19 were associated with lower workplace satisfaction, higher psychological distress and turnover intention of frontline nurses [57]. There were higher levels of fear found in staff with no COVID-19 related training [57]. Continuous learning is a coping strategy to mitigate moral distress [58].

According to previous reviews of the literature, the lack of material and human resources also had a negative impact on the mental well-being of healthcare staff [59, 60]. This lack of resources is well documented [41, 52, 59]. Temeng et al. identified different organisational strategies to support nurses during epidemiological emergencies including ongoing training; improving working conditions; frequent communication from leaders; and counselling sessions [51]. Spain requires a strategic back-up plan and must also improve working conditions and methods for hiring medical personnel [41].

Finally, research confirms that there are structural barriers to nurses accessing leadership positions [33, 61] as well as positions that can influence policymaking [12, 33, 62]. In one Spanish AC, a current ruling prevents nurses from holding first-level healthcare management roles (this ruling is currently being appealed in the Supreme Court) [63], while other ACs have rulings in force that benefit nurses in this regard [64]. Medical dominance continues to be an obstacle to nurse involvement in decision-making processes [35, 61, 62]. Nursing as a discipline is considered inferior [12, 59, 62], frequently deemed as subordinate to physicians [33]. There is also a persisting gender bias. Globally, female health workers are underrepresented in senior official roles such as management, leadership and governance [65]. Gender bias and a perceived subordinate position of nurses have been documented as a barrier to advancing nurse leadership on an international level [61]. In a survey carried out in Spain, a higher percentage of male nurses were found to hold managerial roles than female nurses [66], when less than 20% of Spanish nurses are men [67]. This is the ‘glass escalator' phenomenon, which concerns men working in primarily female-dominated fields [54].

The beneficial impact of nursing has been documented across different settings (communities, clinical care and public health), finding improved quality of care and better health results for patients, largely due to the fact that nurses are permitted to lead and shape health services [12]. Leadership positions for nursing workforce governance must be promoted, and nurses must be encouraged to participate in healthcare policy design processes. To do this, Spain must formally implement the role of GCNO within the Ministry of Health (with the corresponding role, competencies and budget) as well as in other senior official roles in executive and political spheres. Investing in nursing has a ‘triple impact'—it will promote health, gender equality and economic growth [12].

### 4.1. Study Limitations

This study presents several limitations that should be kept in mind when applying the results to international contexts, especially considering the decentralised healthcare system in Spain. In addition, the representativeness of our data may be affected, as existing differences between ACs may limit the generalisability of findings on a national scale. The study includes informants from 13 of the 17 ACs, providing broad, but not total, coverage of Spanish territory. Even so, the identified facilitators and barriers may be transferable to other decentralised healthcare systems facing similar governance challenges.

Another limitation is the scarce transparency observed on websites of healthcare institutions, which made the identification and subsequent contacting of potential eligible ENLs more difficult. Regarding participant profiles, 75% of participants were women, a figure similar to the percentage of women working in the nursing profession in Spain (84.2%) [67]. However, as there are no official data on gender distribution of the senior official roles analysed, it is not possible to determine whether any gender has been underrepresented in the sample.

Finally, there is a potential self-reporting bias inherent to interview-based studies.

## 5. Conclusions

This study offers original findings on the influence of Spanish ENLs on political decision-making during the COVID-19 pandemic. In addition, it examines the ability of these leader nurses to influence healthcare policymaking. Furthermore, it identifies facilitators and barriers influencing both decision-making processes and the implementation of ENL decisions. To the best of our knowledge, this work offers the first description of influencing factors on nurse leadership on a national scale in Spain.

Understanding current involvement of Spanish nurses in healthcare policymaking, as well as the factors implicated in decision-making processes, is key to designing strategies that promote nurse involvement on both an executive and policymaking level.

As such, in Spain, there is a need to promote legislative reform that facilitates nurse access to executive positions of authority, as well as a need for full formal implementation of the GCNO figure. Simultaneously, there is a need for governmental strategies that encourage nurse leadership, eliminating the identified barriers, countering prejudice and gender bias, and recognising the value of the nursing discipline based on its well-documented contribution during the COVID-19 healthcare crisis. To this end, incorporating ENLs into national and regional crisis committees is required to strengthen evidence-based policymaking. The study also identifies several areas for improvement that must be addressed by the Spanish National Health System as a whole, with the aim of solidifying its preparedness for future healthcare emergencies in particular, developing a National Health Emergency Preparedness Plan with predefined contingency protocols. Finally, this research underscores the need to incorporate specific training programmes on nurse leadership, including essential soft skills, both in undergraduate degree programmes and in specialist postgraduate education. There is also a need to standardise ENL leadership and crisis management training through accredited postgraduate programmes.

## Figures and Tables

**Table 1:** Sample inclusion and exclusion criteria.

Inclusion criteria	Exclusion criteria
Assistant Chief Nurse Officer^∗^ of a healthcare organisation	Nurses with no organisational responsibility
Chief Nurse Officer of a healthcare organisation	Ward managers
Chief Executive Officer	Nurses in private healthcare hospitals
Chief Nurse Executive in the autonomous government	
Chief Nurse Executive in the autonomous health system	

^∗^Positions below CNO within the healthcare organisation's executive committee.

**Table 2:** Sociodemographic data.

Category	Demographic subcategory	*N*	%
Sex	Male	6	25
	Female	18	75

Age	25–34	1	4.1
	35–44	3	12.5
	45–54	8	33.3
	55–64	9	37.5
	65 or more	3	12.5

Highest level of education	Degree	4	16.6
	Second university degree	1	4.1
	Master's degree	14	58.3
	Clinical Nurse Sspecialist	2	8.3
	PhD	3	12.5

Time in current role	1 to 5 years	15	62.5
	6 to 10 years	7	29.1
	11 to 15 years	2	8.3

Time in a leadership role	1 to 9 years	6	25
	10 to 19 years	8	33.3
	20 to 29 years	6	25
	30 to 39 years	2	8.3
	40 or more	2	8.3

Executive management	CNE autonomous health policy	8	33.3
	CEO healthcare organisation	4	16.7
	CNO healthcare organisation	9	37.5
	Assistant CNO healthcare organisation	3	12.5

**Table 3:** Additional quotes.

Code	Quote
Description of political participation	In that moment, I felt like I was at the top, so, yes, above us is the interterritorial board [meeting between the health minister of ACs and the ministry of health] who made some decisions that we complied with, obviously, but below them, I felt, at the time, that at least people listened to suggestions I had. M13
	Nursing was put into the public eye, people have seen everything we do and that we're able to lead, so they need to invest [resources] and believe in us. Visibility is temporary and fleeting if we don't get put into positions of political decision-making [authority]. F10

Opinion on CNE role	It was always questioned why there had to be a specific Chief Nurse Executive and then another Chief Executive for the rest of the staff, and the argument ‘because there are more of us' etc. never carried enough weight. M13
	There's a central services manager who says ‘I manage healthcare and nursing, everyone. I cover everybody.' And I say yes, sure, but [you're] a doctor, you don't see [things] from my perspective. F23
	[There's a] lack of input from upper management on care, unifying actions and improvement efforts. A management team [a CNE in MoHAC] would have had some kind of response, at least we'd have a clear point of reference. F8

CNO's political influence	I had to make important strategic, tactical and organisational decisions in my department. I took part in several managerial committees alongside central healthcare services and the ministry of health of AC. M7
	The [health] minister [of AC] was the type of person that he knew everyone […]. The [health] minister [of AC], and another person, have always been very open to discussion and to listening to you. I mean, I didn't take it as something forced [on me], I also thought that I could take it on […] I took it as a way of putting faith in me, because they knew I could do it. M6

Political pressure on management	Any decisions that are made are always agreed with the directorate General, [political position below AC minister of health] sometimes agreed and other times imposed, because they're part of a strategic approach. F17
	If I were the one to have had to make the decision, I definitely wouldn't have taken it [banning family visits], I wouldn't have done it that way. But I suppose we had to stick to a set of rules. F1

Response coordination	The ministry of health [of AC] and the [Autonomous] community health service, [in a] managerial capacity, we were two parallel worlds. Honestly, [the pandemic] really forced us into action and we've formed close-knit relationships because we had to work hand in hand […]. At first it was so difficult because each side did their own thing, one side said one thing and the other side did another. M6
	We set up a joint information system that didn't exist before, between education and healthcare, because we didn't have access to their information system and we created a sort of bridge where the COVID officer in the school would record the child's details, we'd then be able to access the details and we'd call their parents. So, case management and the initial clinical response was done by nursing. F1

Contingency plan	We [had no previous contingency plans], we made them because they involved many different variables […] yes, we were already familiar with certain PPE because isolating ill patients was nothing new […]. But this required us to be clear: Which areas, where did they go out, where did they come in, […] it caused a change in the structure and organisation of our care units, and in the structure and organisation of the primary care centres [themselves]. F2
	Setting up the contingency plan because we were overrun. I think that's what put emergency services in such a tight spot, [they'd say to me:] ‘they said that at the referring hospital they didn't have any respirators, at the hospital […] they also have no respirators, and I have three guys intubated outside. What do we do?'. That contingency plan, for me, that was the most impactful decision […]. Luckily, thank God, we didn't have to activate it, a respirator always appeared […]. But the fact we had to sign that document and keep it aside just in case we needed it, that was really hard. M3

State of alarm	The state of alarm, it covers you somewhat for making certain decisions that you wouldn't normally be able to make with no state of alarm. F18
	In that moment, every barrier was lifted, […] there were no more bureaucratic hurdles. F1

New technologies and early warning systems	Yes, we did have vital IT systems, […] they've been spectacular […]. It's not easy, we're talking another level of big data, […]. Of course the IT systems were important. F17
	They set up early warning systems so that every COVID case that came from outside and went through the Emergency department and/or ones that started in hospital would flag on the early warning systems. They had us monitor exactly what was going on, every hour, on the dot. [The systems] are still active. F5

Communication tools	As I had a really good relationship with [healthcare organisation X], whenever I found an issue I would quickly let them know so that it didn't happen again, and vice versa […]. I shared things with them because of our good relationship. F20
	PPE training, informational posters, websites where we could upload all of the documentation, sharing with our networks, etc. F17
	Using the early alert systems […] we had constant access to information, […] that information had to be shared around because the organisation had to know what was going on, so everyone knew about changes in decisions being made, which was one of the main complaints we found from our analysis in the first wave. F5

Positive acceptance of the decisions	We spoke with them often, having meetings with them and it was us who explained to them why certain decisions had been made, and we'd explain it and they'd understand. F24
	I think decisions were accepted on the whole. Some perhaps not, but generally speaking, they were accepted and had decent outcomes […]. Many [staff members] noted that some of the decisions that were made helped put them at ease, they felt like they had been listened to. M13

Training and previous experience	I had the education behind me, I did the ‘executive MBA' with a specialisation in financial management, I was in charge of everything to do with operations and overseeing management and budgeting in [the healthcare organisation]. [I had] quite a lot of strategic influence when it came to decision-making. In fact, our CEO was absent for a time due to some health issues, and I stepped in for him. M3
	A plus for us was that we'd worked with disaster planning quite a lot, […] it's something we've been used to dealing with for many years. There were several experts in disaster management here […]. So we used those methods we normally apply when reallocating resources. F4
	At first, I didn't quite understand the mid- to long-term impact of the pandemic, but my crisis and emergency management mindset allowed me to get up to speed with this healthcare crisis that was unfolding faster than other executives and managers in the health service. M7

Recognition of role	I Felt strongly supported by my superiors and my staff […] [I also] felt that the other chief officers really rallied around me. M7

Presence of nurses in political positions	I think it does make it easier [when the politician in charge is a nurse] because there's a certain sense of corporatism and nursing needs that. Nursing needs it because corporatism is what has held us back from these kinds of roles. But I don't think you can fulfil the role any better or worse by simply being a nurse. Because [it's not the case for] the ministry [of health of AC], nor the CEO, nor for me: if I'm a complete disaster and I do things terribly, it's not the fault of nurses as a whole. But in the same vein, if I do things well, it's also not down to me being a nurse. M3

Staff fear and fatigue	Managing fear […] the people that were […] treating COVID patients, they were the ones who showed the least fear. Because I suppose they were so deep in the situation […] as you got further and further away from these services […] fear would bubble up so easily […] really very complex situations […]. Managing that fear was one of the hardest things for me […]. We took people out of theatres, especially to the non-COVID ICUs […] because it's not so far off, really, in terms of knowledge and skills. But there was one time in particular where they refused […] there were people who […] weren't willing to do it because of the fear[…].There's a lack of motivation right now that I think has come from fatigue. F4
	There was a lot of fear at first. We had to manage [masks] really well because people took them home for their children, their family. Because there weren't any on the market, there weren't any in pharmacies. Fear is what made many staff members take them [home]. F18
	There was an emotional wearing down among the nursing executive team, there was more aggression, less patience, and I think that's a consequence of the tension that existed during COVID. […]. From a nursing perspective, there's lots of absent staff, and there are no measures in place to handle that, that fatigue among staff, […] of how we hold on to talent, how we stop people from leaving the profession. F8

Lack of resources	You start to see misuse of resources; another is when there starts to be not enough staff for the operations we put in place. F9
	Not having enough trained staff, I had no critical care nurses and had to ask the Autonomous community [government for help]. F5

Unpopular decisions	In the end, almost everyone agreed, aside from [occupational risk] prevention [department]. You know that it's a critical area and well, oftentimes the decisions were left to people in executive roles, but it was basically unanimous. F5
	I did have issues when it came to family members [related to visit ban], the[Occupational risk] prevention service and the head of infectious diseases became very strict. I Wanted to find some alternatives […] but all they were concerned about was preventing contamination, not spreading the infection, and the person [human being concept] wasn't even considered, in every sense of the word. The disease was the priority. F8
	Nurses who aren't happy with how it was managed. I Went to the units and listened to them and […] they didn't feel cared for in many of the units. We made decisions because we thought they were for the best and they didn't see it that way, they would have done it differently. So, yeah, it's unfortunate when they don't agree. F23

Communication barriers	In the first month, I was learning more and more every day. A different strategy would come in from the ministry each day, they'd change strategies so often, what you could do before, now you can't do it anymore. So you were chopping and changing every day […] you had to be constantly adapting your approach. F18
	Like with the [preparation of] vaccines, trying to do everything so quickly, at first it was don't shake it, then if you shake it you could later end up… it was a constant barrage of new information to keep up with and obviously this impacted decision-making because evidence was also constantly changing and you had to reframe things and readjust (…) based on new evidence, you have to evaluate and then modify. F2
	A lot of information that I had from the get-go, it surprised me that it wasn't shared in strategic areas or that it was shared extremely late, or that it wasn't shared equally. F20
	We had a lot of pressure on us from people and from the press. M13

Hiring regulations	I didn't have [enough] qualified staff in the ICU […] [I asked] for a certain percentage of [ICU] beds [that the hospital had to add] to come with staff with [previous ICU] training. I had many arguments with [human resources] and with the chief officers […] they have very obsolete [processes]. F20
	We have specific job banks for staff who are trained in that specialism. […] our bank staff were very limited […]. For example, for staff in the ICU, it was a little like looking in other departments to find staff that could handle working there. There were times during contract renewals where we would say, ‘please don't change [the staff], give them another three months [on their contract] […] and we extended everyone's contracts but then when 30th September came around, we were short staffed in the ICU. It's also kind of down to staff anger. F17

Not having the authority to make decisions	The [autonomous] community health service, in this case, is a rhetorical figure, because it doesn't have any kind of power, which is something I realised […]. When I took the initiative to present that protocol, you realise you don't have any power […]. Had I been the CEO of the health service, I definitely would have done things differently. M6
	The move to self-testing took a lot to convince them […] the ministry [MoHAC], they said that self-testing wasn't valid […] because it could give a false negative if the person didn't do the test properly. You don't say? F5
	In the end, when it comes down to it, since there were other people that didn't quite see it, well that meant decisions couldn't be made in some areas. M7

Negative recognition of the executive committee	Yes, even though you're completely right in what you're saying, some people just assume you're not in the position to tell them how they should be doing things, [even if] you have more personal and professional experience than them, because he is a general director [politician of MoHAC, equivalent to undersecretary of health]. F20

Being a nurse	I had never felt discrimination in my career until this period of time, […] perhaps in some areas I've not felt as discriminated against, perhaps it's also because I've come to the conclusion that, being a man […] And in my time [as ENL], I've felt that discrimination against nurses. M6
	[During] the pandemic […] our managers, CNOs, CEOs, ministries [of AC] realised just how important nurses were, but since the pandemic, I'm convinced that nurses haven't been able to make the most of that realisation. Once again, we're back to, ‘well, now that's over, let's go back into our usual box'. When the place we had before wasn't what we were due. F2
	I noticed big differences when it came to doctors, as if they were wrapped in cotton wool and nurses were there to be constantly mistreated. [For human resources] it was only a problem when it was to do with nurses, not doctors. F20

Female gender	The consequence ended up being my dismissal. That's where I really got that man versus woman, doctor versus nurse feeling. F20

## Data Availability

The data supporting the findings of this study are held by the first author and may be made available upon reasonable and justified request, subject to compliance with ethical guidelines, confidentiality agreements and applicable data protection regulations.

## Supporting Information

The interview script and the completed SRQR [30] checklist can be found in the supporting file.
