# Supplementary material for: Political Influence, Facilitators and Barriers in the Decision-Making Processes of Executive Nurse Leaders During the COVID-19 Pandemic in Spain: An Ethnographic Study
Source: J Nurs Manag. 2025 Nov 4;2025:4761787. doi: 10.1155/jonm/4761787 (PMC12605871; doi:10.1155/jonm/4761787)
Supplement: Supporting Information — Additional supporting information can be found online in the Supporting Information section. [file 4761787.f1.docx]

**Interview script**

1. Describe in brief your role in your institution during the COVID-19 pandemic.
2. Give a brief summary of your involvement in decision making before the pandemic.
3. List the 3 most frequent tasks related to decision-making that you performed during the COVID-19 pandemic.
4. Were you able to get a general understanding of the impact that the COVID-19 pandemic would have play out from the beginning? Did you perceive the workplace issues related to the COVID-19 pandemic from the beginning?
5. List 3 trigger factors that made you clear you had to make decisions during the COVID-19 pandemic.
6. Were you aware of the consequences of the decisions you made during the COVID – 19 pandemic?
7. List up to 3 most relevant decisions you had to make during the COVID-19 pandemic.
8. For each of these 3 decisions, how would you describe them? In what way (how) was it a new/ready-made decision? If it was an imposed decision, who or what imposed that decision?
9. If applicable, did you have any concerns when making decisions during COVID - 19? Can you describe these concerns?
10. Do you think that having had a general understanding of the COVID-19 pandemic had influenced your decision-making? (If answered yes to Question #5) OR- Do you think that the lack of a general understanding of the COVID-19 pandemic had influenced our decision-making? (If answered no to Question #5)
11. Were there some decisions you could not make? Please, further elaborate on the obstacle that hindered your decisions.
12. Could you describe the criteria you used to prioritize your decisions?
13. Did you feel that you had the authority to make decisions you have made? Do you perceive that you were considered by others to be in the right role to make decisions?
14. Did you feel confident in making decisions you have made? Do you perceive that you were considered confident enough by others to make decisions?
15. Did the decisions you made result in positive outcomes? Would you, in hindsight, make the same decisions again? Did these decisions meet your expectations?
16. Did you have to readjust your decisions? What contributed to these readjustments? Which decisions did you have to readjust and why did you have to do so?
17. List up to 3 consequences of your decisions you are more satisfied with. Why do these decisions make you satisfied? What impact on your institution society did they have?
18. List up to 3 consequences of your decisions you are less satisfied with. Why do these decisions make you unsatisfied?
19. What differences have you found in your decision-making role compared to the pre-pandemic situation? Has the degree of your involvement in decision-making during the pandemic changed in some ways?
20. Is there anything else you would like to add?

**Compliance checklist SRQR [O’Brien et al. 2014]**

| No | Item | Verification |
| --- | --- | --- |
| Title and abstract | | |
| 1 | Title | Yes |
| 2 | Abstract | Yes |
| Introduction | | |
| 3 | Problem formulation | Yes |
| 4 | Purpose of study | Yes |
| Methods | | |
| 5 | Qualitative approach and research paradigm | Yes |
| 6 | Researcher characteristics and reflexivity | No |
| 7 | Context | Yes |
| 8 | Sampling strategy | Yes |
| 9 | Ethical issues pertaining to human subjects | Yes |
| 10 | Data collection methods | Yes |
| 11 | Data collection instruments and technologies | Yes |
| 12 | Units of study | Yes |
| 13 | Data processing | Yes |
| 14 | Data analysis | Yes |
| 15 | Techniques to enhance trustworthiness | Yes |
| Results/findings | | |
| 16 | Synthesis and interpretation | Yes |
| 17 | Links to empirical data | Yes |
| Discussion | | |
| 18 | Integration with prior work, implications, transferability and contribution to the field | Yes |
| 19 | Limitations | Yes |
| Other | | |
| 20 | Conflicts of interest | Yes |
| 21 | Funding | Yes |
